# The hidden perils of read mapping as a quality assessment tool in genome sequencing

**DOI:** 10.1038/srep43149

**Published:** 2017-02-22

**Authors:** B. Lehri, A. M. Seddon, A. V. Karlyshev

**Affiliations:** 1 School of Life Sciences, Pharmacy and Chemistry, SEC Faculty, Kingston University, Kingston upon Thames, KT1 2EE, UK.

## Abstract

This article provides a comparative analysis of the various methods of genome sequencing focusing on verification of the assembly quality. The results of a comparative assessment of various *de novo* assembly tools, as well as sequencing technologies, are presented using a recently completed sequence of the genome of *Lactobacillus fermentum* 3872. In particular, quality of assemblies is assessed by using CLC Genomics Workbench read mapping and Optical mapping developed by OpGen. Over-extension of contigs without prior knowledge of contig location can lead to misassembled contigs, even when commonly used quality indicators such as read mapping suggest that a contig is well assembled. Precautions must also be undertaken when using long read sequencing technology, which may also lead to misassembled contigs.

Determination of complete sequences of genomes is paramount for understanding an organism’s biology and function. Despite the exponential accumulation of sequencing data, the vast majority of genomes deposited in GenBank represent only ‘draft’ or incomplete versions. Even the genomes of relatively small organisms such as bacteria (up to 10 million bases) are usually submitted as draft assemblies^1^, and those present as complete genomes are often derived from the previously sequenced genomes of very closely related strains of the same species. One example is a large number of complete sequences of different versions of the genome of *Campylobacter jejuni* strain NCTC 11168^2^. Derivation of a complete genome sequence of a distantly related species represents a challenging task. The problem stems from the lack of a universal and reliable tool that would allow automatic contig assembly, particularly with sequences containing long repeats. There are limited tools for validation of the quality of the contigs, and undetected errors may also contribute to the problems. The draft genome sequences are produced by an automatic *de novo* assembly of short reads generated by using different whole genome sequencing technologies, such as e.g. Ion Torrent PGM. Automatic *de novo* assembly of a large number of short reads is relatively cheap and often provides good genome coverage but usually results in a number of disconnected contigs due to the presence of repetitive sequences. Given that the low quality reads are removed, the high sequence coverage obtained from short reads can be useful in identifying nucleotide-level variants such as indels or SNPs. On the other hand, long read technology tends to produce low quality reads with low sequence coverage, although the reads can span across repetitive elements. Optical mapping is an additional tool which allows arrangement of contigs on the chromosome and an estimation of gap sizes and their positions. A combination of several methods used for genome sequencing and assembly, which is named a hybrid approach, can potentially lead to a high quality genome assembly^3^.

However, both with separate contigs and final genome sequence there remains a problem with assembly quality validation. Misassembly can affect various downstream applications including comparative genomic analysis. If misassembled genomes are used as references for assembly of other (similar) genomes, the errors could be carried over to the sequences being assembled. Although some bioinformatics approaches to be used for the identification of assembly errors have been suggested^4^ they are normally based on comparison with previously available data. However, in many cases an independent validation tool is required.

In this study we provide an example of sequence misassemblies generated by *de novo* assembly and contig extension approaches using unpaired reads. In addition, we demonstrate that, despite being widely used, the assembly quality verification by read mapping could be misleading. Finally, we suggest a way of generating reliable genome assemblies by utilising different approaches and assembly verification tools.

## Materials and Methods

### Sequencing

Total genomic DNA from *L. fermentum* strain 3872 was extracted using the Gentra Puregene Yeast/Bact. kit (Qiagen). DNA quality was assessed using Nanoview photometer, Qubit 2.0 fluorometer (Thermo Fisher Scientific/Life Technologies) and gel electrophoresis. Three sequencing runs were conducted using Ion Torrent PGM, Ion PGM sequencing kit 400 bp, Ion PGM template OT2 system and 314v2 chip, which generated unpaired reads. Long sequencing reads were generated using a PacBio RSII sequencer with the P6-C4 sequencing chemistry and a single SMRT-Cell. The numbers of sequencing reads in PacBio run and runs 1, 2 and 3 (Ion Torrent) were 74,588; 387,040; 478,928 and 422,749 (respectively), with the mean read sizes for being 7176, 288, 291 and 274 bases (respectively).

Optical map was generated with the Argus Optical mapping system by OpGen and restriction enzyme *Spe*I. MIRA and SPAdes *de novo* assemblies were run on the Ion Torrent server which used parameters specifically optimised for the Ion Torrent reads. CLC Genomics Workbench (CLC GWB) assembly was conducted using the minimum contig size of 1000 bases. Default CLC read mapping parameters were: mismatch cost 2, insertion cost 3, deletion cost 3, length fraction 0.5 and similarity fraction 0.8. A length fraction of 0.8 and a similarity fraction of 0.9 was chosen for stringent read mapping parameters, a length fraction of 0.9 and a similarity fraction of 1 was chosen for very stringent read mapping parameters. For both stringent and very stringent read mappings other parameters were kept the same as the default.

## Results

Unpaired sequencing reads produced by the Ion Torrent PGM were assembled *de novo* using MIRA 3.4.2.0 and SPAdes V3.5.0 5 plugins of the Ion Torrent server, as well as CLC GWB assembler (Table 1). The MIRA, SPAdes, and CLC GWB generated contigs were combined using CISA contigs integrator^6^. This generated 43 contigs with 88.16% of read nucleotides mapped. CLC GWB with default read mapping parameters was used to identify contigs that were misassembled by looking for unaligned ends, gaps and low coverage areas. Misassembled contigs were split at regions where the read mapping conflicted with the assembly, generating 48 contigs with the total size of 2,497,362 nt (N50 = 60,618 nt). Sequences at the ends of the contigs were analysed using NCBI BlastN and a non-redundant database. If a match was found the section with the highest hit was extended and added to a respective contig. The extended sequence was then verified using read mapping. Some of these new contigs could also be merged by using non-repetitive overlaps at their ends, as well as *de novo* assemblies of the unmapped reads. This allowed the reduction of the number of the contigs to 18 with the total assembly size of 2.5 Mb (N50 = 310,835 nt). This also increased read mapping coverage (99.65% nucleotides of reads mapped).

**Figure 1:**
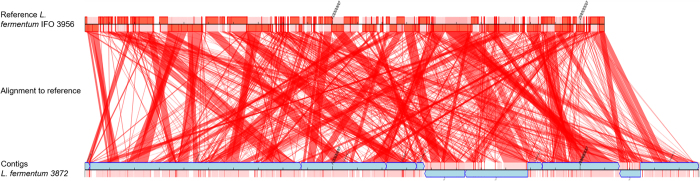
Contiguator scaffold alignment of *L. fermentum* 3872 original assembly (18 contigs, see text) to reference genome sequence of *L. fermentum* IFO 3956. BlastN with default parameters was used. Only the contigs (blue) that mapped to the reference genome are indicated. The red lines indicate regions of the query genome where there is a BlastN hit to the reference genome.

One other option of arranging contigs in order and joining them together is to align them to a known complete genome sequence of another closely related strain of the species. This approach is based on the assumption that, despite strain to strain variation such as the presence of single nucleotide variations and indels, the overall genome organisation would be similar (although this may not be the case for some species). It is also based on the assumption that the reference genome sequence does not have significant errors. For that purpose, we aligned the contigs to a reference genome of *L. fermentum* IFO 3956 using Contiguator software version 2.7.4 7 and BlastN with a cut-off e-value of 1e-20. As seen from mapping shown in Fig. 1, regions within each contig aligned to various locations of the reference genome, which indicated that there was either very low similarity in the genome organisation between the two strains of *L. fermentum,* or that there were assembly errors. The likelihood that assembly errors were the cause of such differences was considered to be low due to good read mapping of sequencing reads to the contigs, demonstrating no low coverage areas and unaligned read ends when using default, stringent and very stringent read parameters.

**Figure 2:**
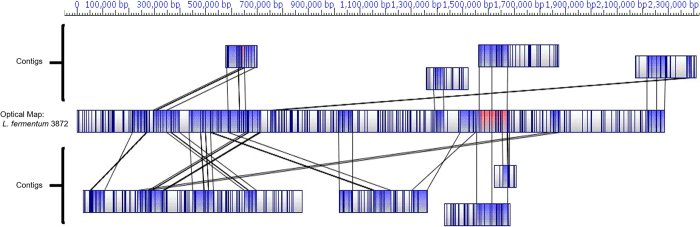
Comparative analysis of optical maps of selected contigs from the original assembly (18 contigs) with that of the optical map of the *L. fermentum* 3872 chromosome. MapSolver tool with default parameters was used. Blue colours specify areas of restriction map similarity; regions coloured in red indicate areas with similar restriction maps at more than one location. Uncoloured regions indicate areas with no similarity.

In order to assess the quality of the assembled contigs an optical map of *L. fermentum* strain 3872 genome was generated. Optical mapping is a sequence independent tool for generation of a restriction map. Contigs were aligned to the optical map using MapSolver software with default alignment parameters. This showed that there were considerable assembly errors, even when the read mapping suggested a well assembled contig (Fig. 2).

**Figure 3:**
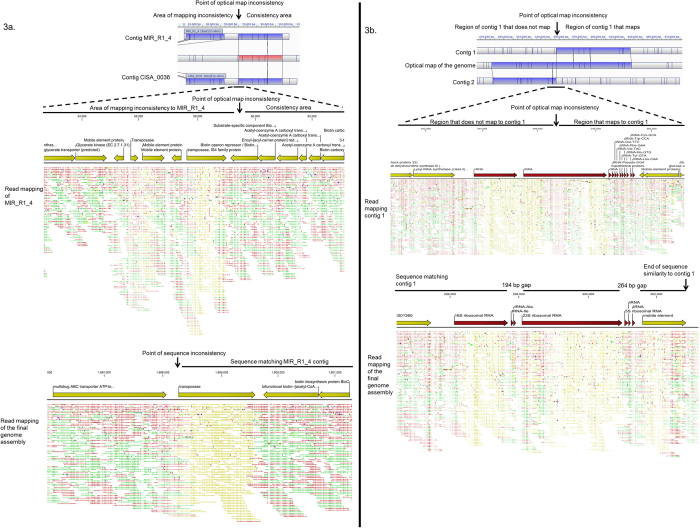
Using read mapping for the identification of the points of misassembly identified by optical mapping. A, misassembled contig 4 from MIRA *de novo* assembly and contig 36 from CISA assembly; B, misassembled contigs 1 and 2 from the 18 contigs assembly. CLC GWB (default parameters) was used as a read mapping tool. Read colours: green, red and yellow indicate forward, complementary and repetitive sequences respectively. Single nucleotide mismatches, which appear as dots, according to the standard CLC GWB colour scheme, represent technical errors produced by the IonTorrent PGM. Due to their relatively rare appearance and irregular positions, these mismatches don’t represent any biological significance, have little influence on the overall read mapping picture and are not relevant to the topic of this study. Reads from all three Ion Torrent runs were used for mapping. MapSolver was used as an optical map alignment tool (default parameters); blue colours specify areas of restriction map similarity and uncoloured regions indicate dissimilar restriction maps.

**Figure 4:**
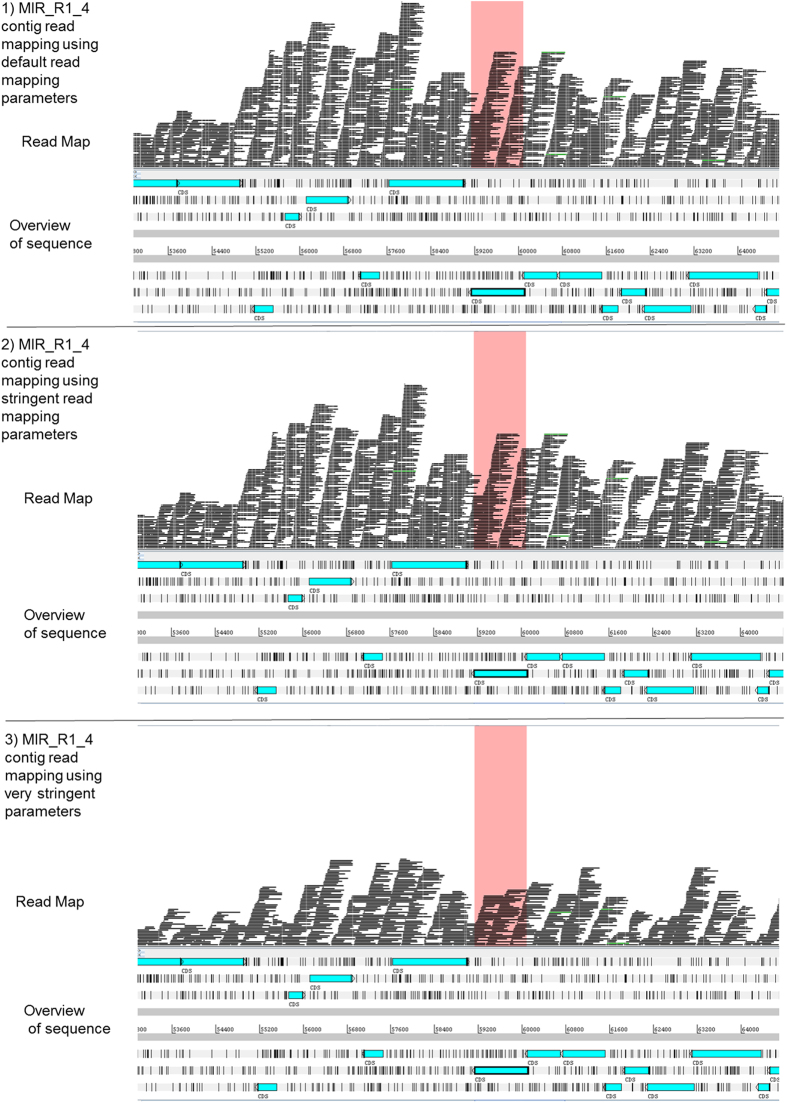
Read Mapping of MIRA run 1 contig 4 (MIR_R1_4) using Ion Torrent reads generated from run 1. The read mapping was conducted using a CLC GWB mapping tool with default, stringent, and very stringent read parameters on. The BAM file generated by read mapping was transferred to ARTEMIS tool for visualisation^18^. The area highlighted in red indicates the unaligned region as shown for this contig in Fig. 3a.

In order to assess whether assembly errors were introduced during the *de novo* assembly process using CLC GWB, MIRA and SPAdes programs, or by CISA contigs integrator, the contigs were aligned with the optical map of the genome (Table 2). Some misassemblies were found to be specific to particular regions of the genome, while others were sequencing run- or software-specific. Assembly errors in original *de novo* contigs were usually transferred to CISA contigs (Fig. 3a), except for one instance where a misassembly was corrected. Despite an increase in sizes of CISA-generated contigs, there was no improvement in alignment of the latter onto the optical map of the genome. Moreover, further contiguation made the assembly considerably worse as none of the final 18 contigs were aligned correctly (Fig. 2). The contigs (Fig. 3a) would not have been considered misassembled if an optical map were not available, as the read mappings of the contigs were good when using default, stringent and very stringent read mapping parameters (Fig. 4). Similar misassembly problems were found for some other contigs, which had been verified by read mapping (Fig. 3b). Therefore, an alternative approach based on long sequencing reads was employed.

Long sequencing reads generated by PacBio RSII sequencing machine were assembled using HGAP3 (hierarchical genome assembly process) pipeline employing CELERA program^8^, allowing the removal of low quality reads and resulting in seven contigs. The largest contig represented the chromosomal sequence of *L. fermentum* 3872 with the size of 2,302,236 nt, exceeding the actual (final) chromosome size. Analysis of this sequence revealed very large redundancies at the ends. Removal of these overlapping regions allowed the construction of a circular sequence of the chromosome 2,297,851 nt.

**Figure 5:**
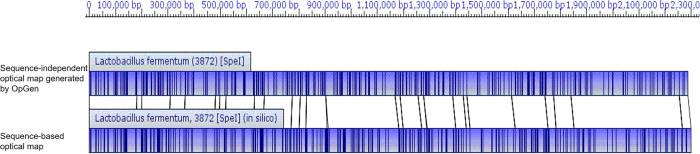
Optical map of the final chromosomal genome sequence of *L. fermentum* 3872. The diagram was generated with MapSolver software (default parameters). The blue coloured and uncoloured areas indicate regions with similar and dissimilar restriction maps respectively.

The *Spe*I restriction map of the final chromosomal assembly was in excellent agreement with the sequence-independent optical map generated by OpGen (Fig. 5). In addition, the assembly revealed excellent coverage with 99.70% of nucleotides of the Ion Torrent nucleotide reads mapped onto it, with unmapped reads being of very small size and low quality.

Despite overall good sequence assembly produced by PacBio (after removal of redundancies at the ends), the sequence had to be verified for potential errors due to relatively low coverage by the long reads (19.47x). This was performed by utilising the data generated by Ion Torrent sequencing. Mapping of short reads to the genome assembly did reveal errors (limited to single base substitutions), which were corrected.

**Figure 6:**
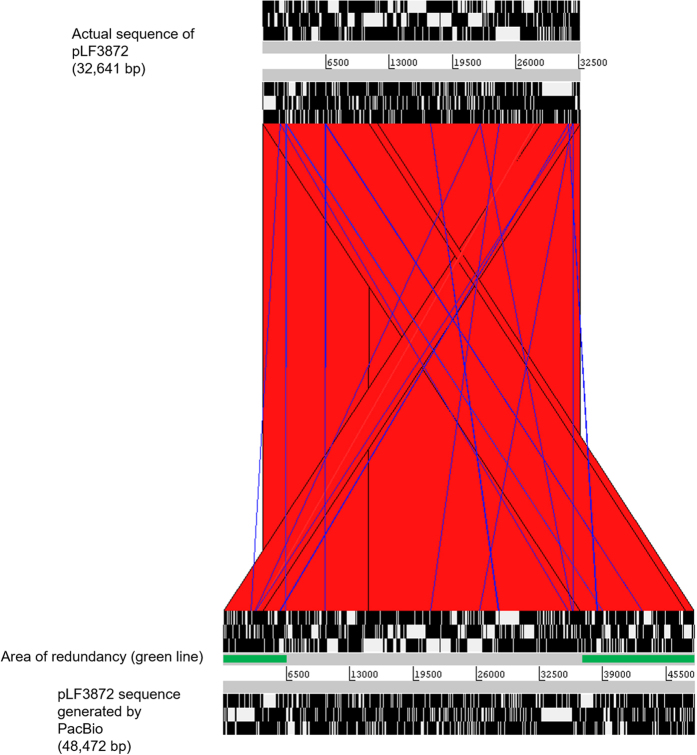
Comparison of Ion Torrent and PacBio RSII generated sequences of plasmid pLF3872 using Artemis Comparison Tool (ACT). Red coloured areas connect similar areas in the sequences.

One of the contigs generated by the PacBio tool was found to represent a plasmid. As the complete sequence of this plasmid had been previously determined and reported^9^, it provided another opportunity to assess the quality of the assembly. As with the chromosome assembly described above, the estimated size of the plasmid was significantly overestimated, i.e. 48,472 nt instead of the actual size of 32,641 nt. The reason for the redundancy was detected by using the ACT comparison tool^10^ which revealed extensive overlapping parts at the ends (Fig. 6). Merging the redundant areas at the ends allowed circularisation of the plasmid sequence making it identical to the one based on Ion Torrent sequencing data and published earlier^9^ (Fig. 6).

The remaining five contigs (with sizes between 14 kb and 18 kb) generated by PacBio represented fragments of the chromosomal sequence, rather than extra-chromosomal DNAs, as was presumed originally. A distinctive feature of these contigs (each represented by just one sequencing read) was the presence of transposon-related or integrase-encoding regions. It appeared that HGAP3 *de novo* assembler erroneously classified these sequences as contigs rather than low quality reads.

The completed genome sequence of *L. fermentum* 3872 (CP011536.1) is 2.33 MB long including plasmid pLF3872 (32,641 bp in length, CP011537.1)^9^,^11^. At the time of preparation of this manuscript, there were only three completed genome sequences of the species present within the GenBank database, with the remaining 19 being only draft genome sequences.

## Discussion

The completion of the *L. fermentum* 3872 genome sequence revealed various problems that could not be detected by using such common assembly quality indicators as read mapping for unpaired reads. With paired end reads there is a lot of bioinformatics support for the improvement of sequence quality and contig gap closure. For example, ICORN^12^ can be useful for correction of short nucleotide errors representing a sequence, IMAGE^13^ assisting in closing genome sequencing gaps and REAPR^14^, a tool for correcting genome assembly errors. Neither IMAGE nor REAPR require a reference genome. Unfortunately, such tools are not available when utilising unpaired reads generated by Ion Torrent PGM, and thus there is a need for a reference genome. Mate paired reads may also allow the generation of a sequencing scaffold and a better genome assembly when combined with unpaired reads. However, the cost of producing mate pair libraries for the Ion Torrent PGM is higher than long read sequencing using PACBIO RSII, and it also requires considerable bioinformatics analysis.

**Figure 7:**
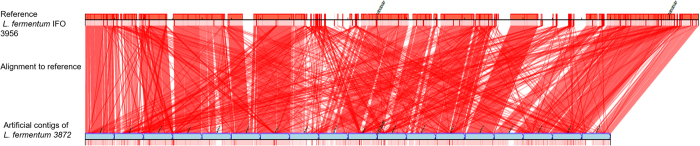
Contiguator scaffold alignment of contigs generated by artificial splitting of the final chromosome assembly of *L. fermentum* 3872 at 100,000 bp intervals to reference genome sequence of *L. fermentum* IFO 3956. BlastN with default parameters was used. Only the contigs (blue) that mapped to the reference genome are indicated. The red lines indicate regions of the query genome where there is a BlastN hit to the reference genome.

Initial assembly verification can be performed by using bioinformatics scaffolding tools such as ABACAS^15^ or Contiguator^7^, which are based on the availability of a closely related complete genome sequences. For example, the Contiguator tool revealed misassembled contigs, which had been previously verified by read mapping. When artificially splitting the final *L. fermentum* 3872 genome sequence at 100,000 bp points, Contiguator showed better alignments to the reference genome (Fig. 7).

A major and the most challenging issue in genome sequencing using traditional short read-based approaches including Ion Torrent technology is joining of *de novo* generated contigs (or closing the gaps). In case the contigs are misassembled, closing of the gaps by PCR and Sanger sequencing could be problematic, particularly when the gap sizes could not be predicted. An alternative approach is to use a single primer PCR technique allowing gradual extension of ends of contig sequences by primers walking^16^.

Analysis of *de novo* contigs revealed that misassembly mainly occurred at locations containing repetitive sequences, such as transposons and ribosomal RNA encoding genes. In an example shown in Fig. 3 the point of misassembly resides in close proximity to a repetitive element. Since repetitive elements are commonly present at the ends of *de novo* contigs, it would be best not to extend such regions (e.g. by primer walking etc) unless information is known about the gene length and contig position. Due to different algorithms, errors in *de novo* assembled contigs are also dependent on the software used (Fig. 3b).

The application of optical mapping for verification of a genome assembly has a lot of potential in that it can provide quality assessment of an assembly without the use of sequencing data, thus removing sequencing bias. As genome sequence technology is focusing on larger read lengths with low quality base calling abilities, optical maps may be useful tools for assessing the quality of an assembly. Optical maps could also be used to identify reads that are artificially indicated as contigs. Utilisation of restriction mapping during assembly process would allow significant improvement of the assembly accuracy^17^. One obvious disadvantage of the current optical mapping procedure is low resolution and the limitation to large fragments (typically over 30 kb, depending on the genome and restriction enzyme selected).

## Conclusion

In this study we revealed potential hidden issues associated with *de novo* assemblies of short unpaired reads, when the assembly quality relied solely on ‘read mapping’. The latter is a very helpful tool revealing major assembly issues. However, the absence of low coverage areas does not always indicate that the assembly is correct. The issue is particularly important in the absence of high quality complete genomes of closely related strain or species, in which case a sequence similarity-based contig extension may produce misleading results. Optical mapping increases the confidence in a genome sequence assembly by removing sequencing bias and should be used at the final stage of sequencing of large genomes.

## Additional Information

**How to cite this article:** Lehri, B. *et al*. The hidden perils of read mapping as a quality assessment tool in genome sequencing. *Sci. Rep.* **7**, 43149; doi: 10.1038/srep43149 (2017).

**Publisher's note:** Springer Nature remains neutral with regard to jurisdictional claims in published maps and institutional affiliations.

## Figures and Tables

**Table 1:** Summary of the results produced by using *de novo* sequence assembly tools (CLC GWB, MIRA and SPAdes) and CISA Contigs Integrator for three Ion Torrent genome sequencing runs; the results of HGAP3 assembly of PacBio RSII reads are also shown.

Assembly tool	Ion Torrent										PacBio RSII
	Run 1			Run 2			Run 3				
	CLC GWB	MIRA	SPAdes	CLC GWB	MIRA	SPAdes	CLC GWB	MIRA	SPAdes	CISA Contigs Integrator	HGAP3
Number of contigs	162	162	288	152	178	291	180	139	288	43	7
N50 (bp)	26507	37091	31838	28624	32493	32116	20853	44927	36130	60618	2302236
Total assembly (bp)	2103399	232129	218533	210611	231359	218484	207929	232812	218426	2497362	2430608
Largest contig (bp)	89244	153205	123470	89537	124270	123504	55652	126522	123449	152374	2302236

**Table 2:** Summary of Contigs that aligned to the Optical map and the number of misassembled contigs.

	Ion Torrent											PacBio RSII
	Run 1			Run 2			Run 3					
	CLC GWB	MIRA	SPAdes	CLC GWB	MIRA	SPAdes	CLC GWB	MIRA	SPAdes	CISA Contig Integrator	18 Contigs	Final assembly (HGAP3)
Contigs aligned to the Optical Map	9	7	12	8	12	10	5	11	11	12	8	1
Missassembled Contigs	1	2	2	1	1	1	1	4	1	4	8	0
